# Paraspinal Muscle Morphology in Proximal Cervical Spondylotic Amyotrophy

**DOI:** 10.7759/cureus.64255

**Published:** 2024-07-10

**Authors:** Ryosuke Hirota, Hiroyuki Takashima, Makoto Emori, Tsuneo Takebayashi, Atsushi Teramoto

**Affiliations:** 1 Orthopaedic Surgery, Sapporo Medical University, Sapporo, JPN; 2 Health Sciences, Hokkaido University, Sapporo, JPN; 3 Orthopaedic Surgery, Sapporo Maruyama Orthopedic Hospital, Sapporo, JPN

**Keywords:** minimally invasive surgery, selective laminoplasty, proximal cervical spondylotic amyotrophy, trapezius muscle, paraspinal muscle

## Abstract

Study design and objective: This is a retrospective analysis of prospectively collected single-center observational data. The aim is to evaluate atrophy and fatty degeneration rates of cervical spinal muscles in proximal cervical spondylotic amyotrophy (PCSA).

Overview of literature: Proximal cervical spondylotic amyotrophy affects muscles in the upper extremities. In cases that lack improvement with conservative treatment, surgery is recommended. However, preoperative factors associated with poor outcomes remain unclear. We hypothesized that assessing fatty degeneration of the cervical spinal muscles and examining its relationship with functional impairment would help predict postoperative improvement in neurological function.

Methods: This study included 18 patients who underwent PCSA surgery. We performed selective laminoplasty and foraminotomy. Preoperative paraspinal muscle cross-sectional area and fatty degeneration were quantified and correlated with neurological function.

Results: Neurological improvement based on manual muscle testing was observed in 12/18 patients, comparing preoperative, perioperative, and over 12-month postoperative statuses. On the affected side, at the C4/5 level, fatty degeneration was more significant in the trapezius, whereas at the C5/6 level, fatty degeneration was more significant in the splenius capitis and trapezius. The fatty degeneration of the C4/5 and C5/6 trapezius was significantly correlated with preoperative muscle strength and postoperative muscle strength improvement.

Conclusions: The degree of fat infiltration of the muscle correlated with pre- and postoperative muscle strength at the lesion level. Thus, our results suggest a relationship between cervical muscle morphology and the clinical manifestations of PCSA. The marked increase in trapezius fatty infiltration at the C4/5 and C5/6 levels may be a valuable indicator to predict poor improvements in postoperative muscle strength.

## Introduction

Proximal cervical spondylotic amyotrophy (PCSA) represents a specific form of cervical spondylotic myelopathy, distinguished by the weakening and wasting of muscles in the upper limbs (specifically the scapular, deltoid, and biceps brachii muscles), often accompanied by minimal to no sensory loss or difficulty in walking [1]. It is believed that the sites of damage responsible for amyotrophy can be located in the intradural anterior rootlets and anterior horn, indicating a spinal cord type, or in the extradural anterior root, suggesting a nerve root type, or possibly a mixture of these two locations [2]. Tauchi et al. reported the risk factors for poor outcomes following surgical treatment of cervical spondylotic amyotrophy [3]. The symptom duration and preoperative manual muscle testing (MMT) grade were associated with an increased risk of poor surgical outcomes. Furthermore, they advised that patients with PCSA undergo surgery within four months from when symptoms first appear if non-surgical treatments do not yield success [4]. Factors associated with poor outcomes include long symptom duration, older age, and greater preoperative muscle weakness in patients with PCSA [4-6]. Furthermore, there are some reports regarding the relationship between posterior paravertebral muscles and clinical symptoms [7,8]. Individuals with chronic low back pain, various types and alterations of the accessory muscle, impairment in embryological development, infections, and certain disorders such as scoliosis, sciatica, and disc herniation often experience atrophy and fatty infiltration of the paraspinal muscles [9-12]. This study aimed to investigate how preoperative fatty degeneration affects surgical outcomes and elucidate the pathogenesis of PCSA. To our knowledge, this is the first study to examine prognostic factors for PCSA using imaging evaluation.

## Materials and methods

This study, a retrospective analysis of patient records involving human subjects, was approved by the Sapporo Medical University (approval no. 292-182) and adhered to the ethical guidelines set by both the institutional and national research committees, as well as the 1964 Helsinki Declaration and its subsequent updates or equivalent ethical norms. Informed consent was obtained from all the study participants.

Study design and patient population

This study was a retrospective analysis of prospectively collected single-center observational data. It included a total of 18 patients who had posterior decompression surgery and were monitored for over a year from 2012 to 2018 at a single facility (the institution affiliated with the authors). Initially, we screened 20 patients; however, two of them were lost to follow-up and could not be evaluated postoperatively. The mean age of the patients was 59.3 ± 14.4 years. The cohort included 12 males (66.7%), with a mean height of 164.5 ± 8.2 cm and a mean weight of 61.1 ± 4.7 kg. Each patient exhibited unilateral upper extremity amyotrophy but did not suffer from walking impairments or significant sensory deficits. Exclusion criteria included individuals who had previously undergone shoulder cuff surgery or had degenerative neurological conditions such as amyotrophic lateral sclerosis. 

While variations in the axial artery and vein can compress the brachial plexus, leading to thoracic outlet syndrome and resulting in pain in the upper arm and back of the shoulder along with muscle weakness, similar to PCSA [13], these conditions were ruled out in each case through physical examination and additional tests. Despite undergoing conservative treatment for a minimum of three months, none of the patients exhibited any improvement in muscle strength. Among them, the deltoid muscle was impacted in 11 patients, while seven patients experienced issues with their biceps brachii. The average time from awareness of muscle weakness to MRI was 4.7 (two to eight) months. Table 1 summarizes the clinical data of the patients. 

**Table 1:** Patient characteristics MMT: Manual muscle testing

Demographic characteristics	Value
Age at operation (years)	59.3 ± 14.4
Sex: Male (%)	66.7
Height (cm)	164.5 ± 8.2
Weight (kg)	61.1 ± 4.7
Time from awareness of muscle weakness to MRI scan (in months)	4.7 ± 2.6
Clinical characteristics	Value
Preoperative neurological impairment (MMT scale)	4
	4
	7
	3

Assessment of muscle strength 

The strength of the most severely atrophied muscles before and after surgery was assessed through MMT [14]. Surgeons accredited by The Japanese Society for Spine Surgery and Related Research conducted the MMT during the initial medical evaluation and the most recent follow-up. To avoid bias, a spine surgeon different from the operating surgeon assessed the muscle strength.

Prognosis of the responsible lesion level and surgical procedures

In cases of PCSA where the cause is spinal cord injury, the lesion responsible is identified at the C3/4 or C4/5 level, which corresponds to damage in the C5 or C6 myelomeres, respectively. On the other hand, when the root of the issue is nerve root damage, the lesion pinpointed is at the C4/5 or C5/6 level, affecting the C5 or C6 spinal nerve roots, respectively [15]. The lesion level in the cervical spine was determined through neurological evaluations, CT scans following a myelogram and an MRI. An electrophysiological examination was not conducted. It was clinically challenging to distinguish between damage to the spinal cord and nerve root in most patients.

Shiraishi et al. described selective laminoplasty as a conservative posterior decompression technique that spares the deep cervical muscles [11]. This method includes performing a single laminectomy along with partial laminotomies [16,17]. This procedure is minimally invasive, alleviates axial neck pain, and simplifies postoperative management. Selective laminoplasty, which combines a single laminectomy and foraminotomy, allows for the simultaneous decompression of two adjacent spinal cord levels and the affected lower spinal root. This integrative approach of selective laminoplasty and foraminal decompression provides concurrent relief for both the spinal cord and nerve root [18]. The level of the causative lesion was achieved in all cases in this study; therefore, selective laminoplasty, a minimally invasive procedure, was used.

Measurement of cervical muscles

**Figure 1:**
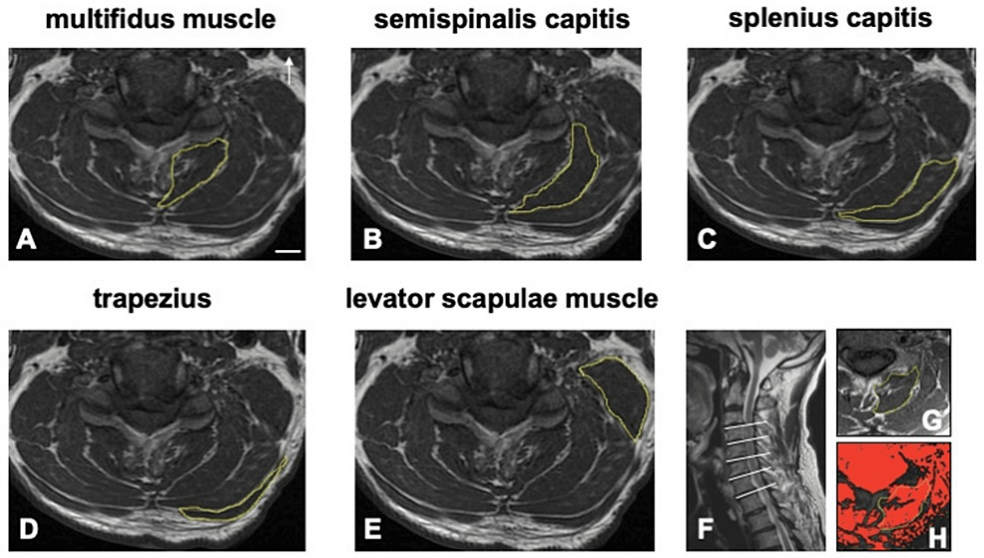
Measurement of cervical muscles A: Cervical multifidus; B: Semispinalis capitis; C: Splenius capitis; D: Trapezius; E: Levator scapulae; F: Center of the disk in the intervertebral space from the C3/4 to the C7/8 level where cervical muscle measurements were bilaterally obtained; G: Muscle morphology evaluated using the percentage of total cross-sectional area (CSA) (muscle size); H: Functional CSA (FCSA)

Cross-sectional area (CSA) and fatty degeneration of the cervical multifidus (Figure 1A), semispinalis capitis (Figure 1B), splenius capitis (Figure 1C), trapezius (Figure 1D), and levator scapulae (Figure 1E) were quantitatively measured. Multiplanar reconstruction (3D MPR) was performed using the SYNAPSE VINCENT version 6.1 (Fujifilm, Tokyo, Japan) to reconstruct image slices perpendicular to the muscle volume. The images were evaluated using T2-weighted axial MR images. The muscle volume and fatty degeneration were quantified and plotted using ImageJ software (National Institutes of Health, Bethesda, MD, USA). Cervical muscle measurements were bilaterally obtained at the center of the disk in the intervertebral space from the C3/4 to the C7/8 level (Figure 1F). Muscle measurements included assessment of total CSA (muscle size) (Figure 1G) and functional CSA (FCSA) (Figure 1H), which represents the fat-free area, and calculation of the FCSA to CSA ratio (FCSA/CSA) as a measure of the fat infiltration of the muscle [7]. The muscle CSA and degree of fatty infiltration were compared between the healthy and affected sides.

Statistical analysis

Paired t-tests were used to compare the difference in CSA and FCSA/CSA between healthy and affected sides at each level. Pearson’s correlation coefficient was used to evaluate the correlation between preoperative muscle weakness, postoperative muscle strength improvement, and fatty degeneration of the paraspinal muscle. A p-value of <0.05 was considered significant. All statistical analyses were conducted using SPSS Statistics version 24.0 (IBM Corporation, Armonk, NY, USA).

## Results

Clinical outcomes

Eleven cases were diagnosed with spinal cord impairment at the C3/4 level and/or nerve root impairment at the C4/5 level. Another seven cases were diagnosed with spinal cord impairment at the C4/5 level and/or nerve root impairment at the C5/6 level. Additionally, four cases were diagnosed with impairments at both levels. Neurological improvement, based on MMT, was observed in 12/18 (66.6%) patients. When comparing the preoperative and >12 (14 to 30) months postoperative statuses, three of four patients classiﬁed as MMT 1 preoperatively improved to MMT 2 (50.0%; 2/4) or MMT 4 (25.0%; 1/4); five of six patients classiﬁed as MMT 2 preoperatively improved to MMT 3 (66.6%; 4/6) or MMT 4 (16.6%; 1/6); two of four patients classiﬁed as MMT 3 preoperatively improved to MMT 4 (25.0%; 1/4) or MMT 5 (25.0%; 1/4); and one of four patients classiﬁed as MMT 4 preoperatively improved to MMT 5 (25.0%; 1/4). No patient had postoperative C5 nerve root palsy or muscle weakness (Table 2).

**Table 2:** MMT of affected muscles before and after surgery MMT: Manual muscle testing

Preoperative MMT		Postoperative MMT					
		1	2	3	4	5	Total
	1	1	2		1		4
	2		1	4	1		6
	3			2	1	1	4
	4				3	1	4
	Total	1	3	6	6	2	18

Preoperative Muscle CSA on the Healthy and Affected Side 

**Figure 2:**
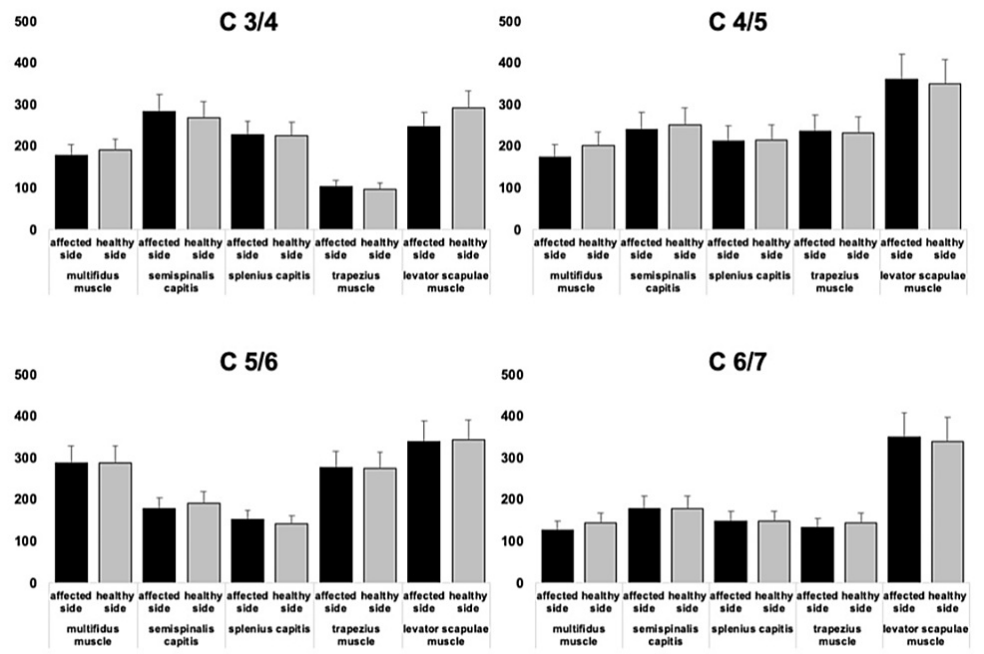
Preoperative muscle CSA on the healthy and affected side There was no significant difference between the CSA of each muscle on each level of the healthy and affected sides. CSA: Cross-sectional area

At each level, there was no significant difference between the CSA of each muscle of the healthy and affected sides (Figure 2).

Preoperative Fatty Degeneration of the Muscle on the Healthy and Affected Side

**Figure 3:**
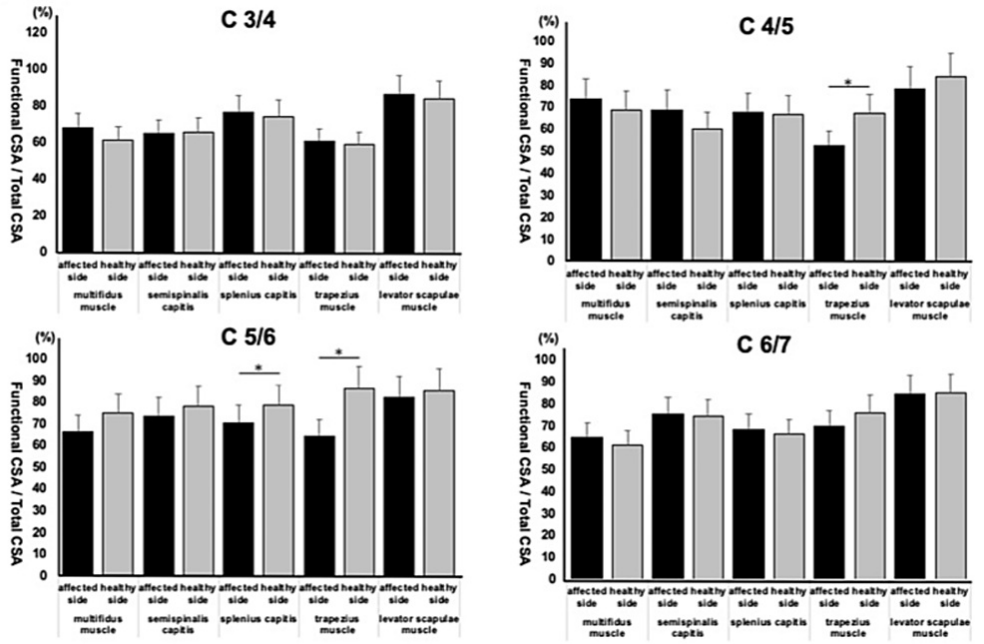
Preoperative fatty degeneration of the muscle on the healthy and affected side *A p-value <0.05 is considered significant. CSA: Cross-sectional area

At the C4/5 level, fatty degeneration was more significant in the trapezius on the affected side (p=0.03). At the C5/6 level, fatty degeneration was more significant in the splenius capitis (p=0.04) and trapezius on the affected side (p=0.03) as shown in Figure 3.

Correlation Between Muscle Parameters and Preoperative Muscle Strength 

**Figure 4:**
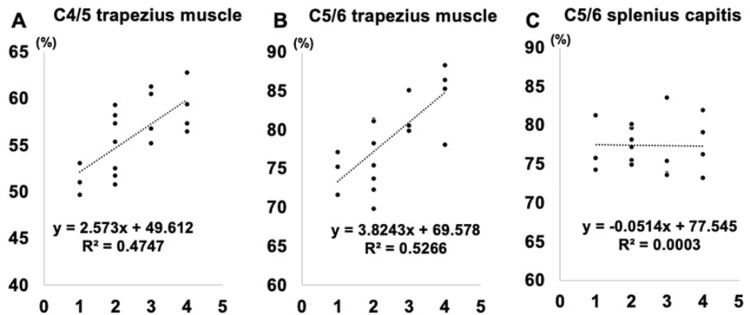
Correlation between muscle parameters and preoperative muscle

Figure 4 shows the correlation between the FCSA/CSA and preoperative muscle strength. The fatty degeneration of the C4/5 and C5/6 trapezius was observed to be significantly correlated with preoperative muscle strength (r = 0.475, p = 0.012, and r = 0.527, p = 0.010, respectively).

Correlation Between Muscle Parameters and Postoperative Muscle Strength Improvement

**Figure 5:**
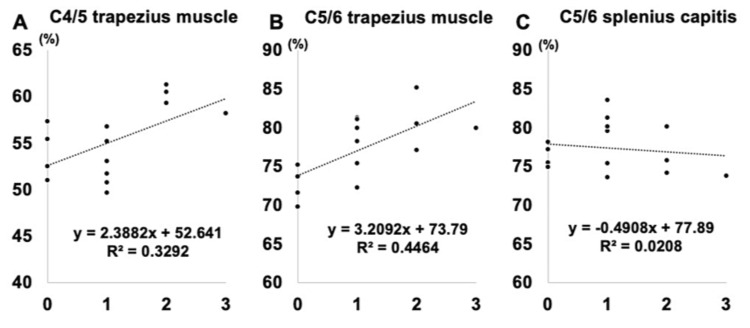
Correlation between muscle parameters and postoperative muscle strength improvement

Figure 5 shows the correlation between the FCSA/CSA and postoperative muscle strength recovery. The fatty degeneration of C4/5 and C5/6 trapezius was observed to be significantly correlated with postoperative muscle strength improvement (r = 0.323, p = 0.038, and r = 0.446, p = 0.351, respectively).

## Discussion

In this study, we evaluated paraspinal muscle morphology in patients with PSCA and correlated it with clinical outcomes. The PCSA was characterized by fatty degeneration of the splenius capitis and trapezius muscles on the affected side. The degree of degeneration of the trapezius muscle was predominantly correlated with muscle strength, both preoperatively and postoperatively. 

Disuse is identified as a potential factor leading to muscle atrophy [19]. A longitudinal investigation among older adults, comparing individuals with and without lower back pain, highlighted a link between the fatty infiltration of trunk muscles and their level of functional activity [20]. Individuals with higher levels of trunk muscle fatty infiltration are at higher risk for exercise-related functional decline over time. Patients experiencing neck pain have been observed to exhibit structural alterations in the deep extensor muscles, including asymmetrical atrophy of CSA, heightened fatty infiltration, an increased ratio of type II fibers, and patterns of delayed activation, in contrast to healthy individuals [21]. In PCSA cases, the overall restriction of movement on the affected side may have also contributed to the development of fatty infiltration in the neck. 

Another possible reason for fatty degeneration of the trapezius muscle is denervation of the accessory nerve. The trapezius muscle is not innervated by spinal myelinated segments at the C5 or C6 levels, nor by nerve roots at the same levels; it is innervated by the accessory nerve. The accessory nerve comprises two distinct parts: the spinal root, which originates from the spinal cord, and the cranial root, which emerges from the brainstem [22]. The accessory nerve branches exit the skull as lateral branches innervating the sternocleidomastoid and trapezius muscles. The accessory nerve originates from a motor neuron nucleus situated laterally in the ventral horn. Its spinal component emerges from the top five or six rootlets, which originate from the spinal nucleus within the lateral gray matter at cervical spinal levels C1 to C5 [23]. The spinal rootlets ascend through the spinal canal and enter the posterior cranial fossa via the foramen magnum, traveling behind the vertebral artery and dorsal to the denticulate ligaments. It then travels to the anterior border of the trapezius muscle, forms a plexus at its surface superior to the clavicle, and enters the deep surface of the trapezius muscle [24]. The primary motor innervation of the trapezius muscle comes from the accessory nerve, although the cervical plexus also contributes. Damage to the accessory nerve is indicated by a constellation of symptoms, including pain in the scapular, shoulder, and neck regions, shoulder weakness and drooping, atrophy of the trapezius, and restricted active abduction in the coronal plane [25]. Electromyography examinations were unavailable for the patients included in this study; however, muscle denervation is common in patients with degenerative cervical myelopathy [26] and could be responsible for the increased fatty infiltration [27]. Skeletal muscle denervation has been observed to cause muscle atrophy, myofiber atrophy, and increased fatty infiltrates [28,29]. These findings suggest that denervation of the accessory nerve and fatty degeneration of the trapezius may occur as neuropathy of the anterior horn of the spinal cord in patients with PCSA. The prognoses were considered poor in cases of such symptoms. 

This study evaluated the morphology of paraspinal muscles in PCSA using MRI; however, it has several limitations. First, it was a retrospective study, and no postoperative MRI evaluations were performed. Second, no electrophysiological studies were performed to assess neurological function, and the study did not include the dominant arm or pain as confounding factors. Third, only a limited number of cases were studied, with only 18 patients included. Future research should consider a larger sample size for more robust data. Additionally, as a future objective, we need to match a healthy control group without PCSA and evaluate the differences in muscle fatty infiltration on both sides using MRI. This will help determine if there is any natural asymmetry.

## Conclusions

This study demonstrates a significant correlation between the degree of fatty infiltration of the trapezius muscle at the C4/5 and C5/6 levels in PCSA and the improvement in muscle strength observed postoperatively. This finding is clinically important as it provides a quantitative measure that may serve as a predictive marker for surgical outcomes in patients with PCSA. Incorporating the assessment of paraspinal muscle morphology via MRI into preoperative evaluations is expected to enhance patient selection for surgery and improve prognostic accuracy. Future studies with larger patient cohorts are warranted to further elucidate the precise relationship between paraspinal muscle condition and neurological function prognosis in PCSA.
